# Clinical and molecular characterization of a transmitted reciprocal translocation t(1;12)(p32.1;q21.3) in a family co-segregating with mental retardation, language delay, and microcephaly

**DOI:** 10.1186/1471-2350-12-70

**Published:** 2011-05-20

**Authors:** Hsiao-Mei Liao, Jye-Siung Fang, Yann-Jang Chen, Kuang-Lun Wu, Kuei-Fang Lee, Chia-Hsiang Chen

**Affiliations:** 1 Institute of Biotechnology, National Tsing-Hua University, Hsinchu, Taiwan; 2 Department of Molecular Biology and Human Genetics, Tzu-Chi University, Hualien, Taiwan; 3 Department of Life Sciences and Institute of Genome Sciences, National Yang-Ming University, Taipei, Taiwan; 4 Department of Teaching and Research, Taipei City Hospital, Taipei, Taiwan; 5 Division of Mental Health and Addiction Medicine, Institute of Population Health Sciences, National Health Research Institutes, Zhunan, Taiwan; 6 Institute of Medical Sciences, Tzu-Chi University, Hualien, Taiwan; 7 Department of Psychiatry, Chang Gung Memorial Hospital at Linkou and Chang Gung University School of Medicine, Taoyuan, Taiwan

## Abstract

**Background:**

Chromosome translocation associated with neurodevelopmental disorders provides an opportunity to identify new disease-associated genes and gain new insight into their function. During chromosome analysis, we identified a reciprocal translocation between chromosomes 1p and 12q, t(1; 12)(p32.1; q21.3), co-segregating with microcephaly, language delay, and severe psychomotor retardation in a mother and her two affected boys.

**Methods:**

Fluorescence in situ hybridization (FISH), long-range PCR, and direct sequencing were used to map the breakpoints on chromosomes 1p and 12q. A reporter gene assay was conducted in human neuroblastoma (SKNSH) and Chinese hamster ovary (CHO) cell lines to assess the functional implication of the fusion sequences between chromosomes 12 and 1.

**Results:**

We determined both breakpoints at the nucleotide level. Neither breakpoint disrupted any known gene directly. The breakpoint on chromosome 1p was located amid a gene-poor region of ~ 1.1 Mb, while the breakpoint on chromosome 12q was located ~ 3.4 kb downstream of the ALX1 gene, a homeobox gene. In the reporter gene assay, we discovered that the fusion sequences construct between chromosomes 12 and 1 had a ~ 1.5 to 2-fold increased reporter gene activity compared with the corresponding normal chromosome 12 sequences construct.

**Conclusion:**

Our findings imply that the translocation may enhance the expression of the ALX1 gene via the position effect and result in the clinical symptoms of this family. Our findings may also expand the clinical phenotype spectrum of ALX1-related human diseases as loss of the ALX1 function was recently reported to result in abnormal craniofacial development.

## Background

Mental retardation (MR) is a childhood-onset neurodevelopmental disorder characterized by a reduced intellectual function that results in learning disability and impaired social adaptation. Approximately 2-3% of the general population is affected with MR; and males are more often affected than females [1]. Genetic defects including gross structural abnormalities of chromosomes, cryptic genomic rearrangements, and monogenic mutations are the leading cause of MR [2,3]. Numerous genes with diversified biological functions have been found to be associated with syndromic and non-syndromic MR; moreover, most of the genetic mutations causing MR are rare, private mutations, indicating that the genetic etiology of MR comprises a variety of highly heterogeneous genetic defects [2,3]. Despite the fact that many genes have been identified as being associated with MR, more MR genes remain to be discovered [2-4].

Chromosomal rearrangements associated with MR may provide an opportunity to discover novel genes associated with this condition. Chromosomal translocations may lead to clinical phenotypes via direct gene disruption, formation of chimera genes, or alteration of the expression of genes near the breakpoint via the position effect [5-7]. Several MR-associated genes have been discovered through mapping of the breakpoints of chromosomal translocations, such as the dedicator of cytokinesis 8 gene (DOCK8) at 9p24 [8]; the potassium large conductance calcium-activated channel, subfamily M, alpha member 1 gene (KCNMA1) at 10q22.3 [9]; the autism susceptibility candidate 2 gene (AUTS2) at 7q11.2 [10]; the oligophrenin 1 gene (OPHN1) at Xq12 [11]; the Cdc42 guanine nucleotide exchange factor (GEF) 9 (ARHGEF9) at Xq11.1 [12]; and the reelin gene (RELN) at 7q22 [13].

As part of serial genetic studies of mental retardation, we detected a reciprocal translocation between chromosome 1p and 12q in the karyotype analysis of a family affected with severe MR, language delay and microcephaly. The translocation was transmitted from the mother to her two boys and co-segregated with the phenotypes. Herein we report the clinical phenotypes and the molecular characterization of the translocation associated with the phenotypes in this family.

## Methods

The Taiwanese family was ascertained through the psychiatric clinic of Tzu-Chi General Hospital, Hualien, Taiwan. The family received medical attention due to the psychomotor retardation of the eldest boy of the family. All family members gave their written consent after all the details of the study were fully explained.

### Karyotype and FISH analysis

Karyotype analysis was performed using the standard GTW-banding method. The breakpoints of chromosomal translocation were investigated using FISH on the metaphase chromosome spreads according to the standard protocol [14].

### Breakpoints identification with PCR and autosequencing

The breakpoint regions were mapped by long-range PCR using the rTth DNA Polymerase XL kit (Applied Biosystems, Foster City, California) according to the manufacturer's protocol. A set of primers were used to PCR amplify the breakpoint regions of the derivative chromosomes 1 and 12, respectively. Aliquots of PCR products that contain the breakpoints were processed using the PCR Pre-Sequencing Kit (USB Cleveland), and subjected to direct sequencing using the ABI Prism™ BigDye™ Terminator Cycle Sequencing Ready Reaction Kit Version 3.1 and the ABI Autosequencer 3730 (Perkin Elmer Applied Biosystems), following the manufacturers' protocols.

### Real-time quantitative PCR (RT-qPCR)

Total RNA was prepared from cell lines and tissues using TRIzol Reagents (Invitrogen Life Technologies, Cartsbad, CA), and cDNA was generated using Superscript II RNase H^- ^Reverse Transcriptase (Invitrogen Life Technologies, Carlsbad, CA). Real-time quantitative PCR (RT-qPCR) was performed using an Applied Biosystems PRISM 7300 Sequence Detection System with continuous SYBR Green detection (Applied Biosystems, Foster City, California). Relative quantification with the standard curve method was used to determine the expression level of the gene of interest. The expression level of each gene was normalized by the expression level of 18S rRNA in each sample, which was measured using Pre-Developed TaqMan Assay Reagents 18S rRNA MGB, according to the manufacturer's protocol (Applied Biosystems, Foster City, California).

### Reporter Gene Activity Assay

An amplicon (1302 bp) comprising 232 bp of chromosome 12 and 1070 bp of chromosome 1 was PCR-amplified from the genomic DNA of affected subjects using primer sets (Chr12-84222865 F-BamHI and Chr1-60785185 R, see Table 1) and cloned into the 3' end of the reporter gene pGL3-control vector (Promega, Madison, WI, USA). The corresponding normal chromosome 12 sequences were also PCR-amplified (1371 bp) from unaffected subjects using primer sets (Chr12-84222865 F-BamHI and Chr12-84224235 R, see Table 1) and cloned into the 3' end of the pGL3-control vector BamHI site for comparison. Transfection of the plasmids containing each of these three different constructs were performed in a SKNSH neuroblastoma cell line and in Chinese hamster ovary cells (CHO) cultured in Minimum Essential Medium (MEM) containing 5% fetal bovine serum in 24-well plates using Lipofetamine™2000 (Invitrogen, California, USA) according to the manufacturer's protocol. At 48 h after transfection, the cells were lysed and the luciferase activities were measured using the Dual-Luciferase Reporter Assay System, according to the manufacturer's instructions (Promega, Madison, WI, USA). The firefly luciferase activity was normalized against the Renilla luciferase activity in each transfection, and reported as the relative luciferase ratio. The differences in gene expression activity between the two constructs were analyzed using a t-test, while differences among the three groups were evaluated by analysis of variance (ANOVA) followed by post-hoc comparison; the p value was set at 0.05.

**Table 1:** Primer sequences and their nucleotide positions on chromosomes 1 and 12 to amplify the breakpoint regions using PCR, and for construction of the expression vectors for the reporter gene assay

primer name	primer location	sequences (5'-3')
Chr1-6	Chr1:61006139-61006163	5'-AGCTTTAACACGGGAGTATAAGGTT-3'
Chr12-5	Chr12:85690331-85690355	5'-CTAATGGGAGGTAGTTGGCTAAGTT-3'
Chr1-6-6	Chr1:61012306-61012330	5'-CTGCAGTCAGACAGATCTAAGATCA-3'
Chr12-5-8	Chr12:85698309-85698333	5'-ACAGTTCTTCTCAAGACAATATGCC-3'
Chr1-Narrow F	Chr1:61013248-61013272	5'-ACTCACTGTGACCCTATGATTTAAG-3'
Chr12-Narrow F	Chr12:85698739-85698763	5'-CCTCCATCTGTTTTATGTAATGCTG-3'
Chr1-Narrow R	Chr1:61013944-61013964	5'-CGGCTGTACACATAGCTACCA-3'
Chr12-Narrow R	Chr12:85699656-85699675	5'-TCATTTATTTGCCCCTTGGA-3'
Chr12-84222865 F-BamHI	Chr12:85698734-85698753	5'-ATAGGATCCCCCAGCCTCCATCTGTTTTA-3
Chr1-60785185 R	Chr1:61012577-61012597	5'-ATAGGATCTTTGGTCAACAATGACTGCAT-3'
Chr12-84224235 R	Chr12:85700085-85700104	5'-ATAGGATCTTGGGGACAAATTCACAGGT-3'

## Results

### Clinical findings

**Figure 1:**
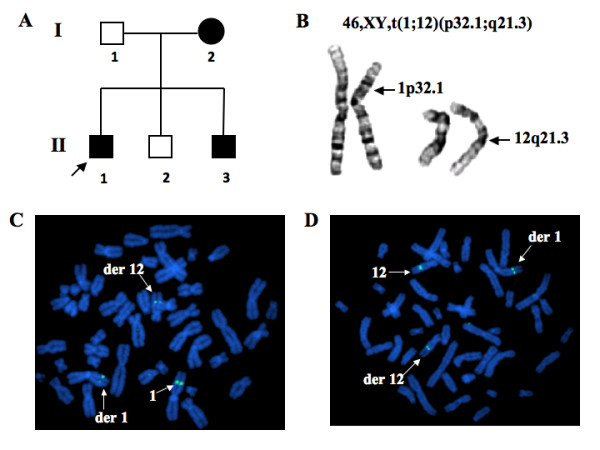
**The results of Karyotype and FISH analysis.** (A) Pedigree of the family with mental retardation, language delay and microcephaly. (B) Standard karyotype analysis revealed a reciprocal translocation between chromosomes 1 and 12, designated t(1, 12)(p32.1; q21.2) in the proband (II-1). (C) FISH analysis showed the chromosome 1 breakpoint may be located within the genomic sequences of the BAC clone RP11-57F6, as the fluorescent signal could be seen on normal chromosome 1, derivative chromosome 1, and derivative chromosome 12. (D) FISH analysis showed that the chromosome 12 breakpoint may be located within the genomic sequences of the BAC clone RP11-259D24, as the fluorescent signal could be seen on normal chromosome 12, derivative chromosome 1, and derivative chromosome 12.

We identified a Taiwanese family affected with severe mental retardation, language delay, and microcephaly in the mother and her two boys (Figure 1A). The proband was a boy who was 6 years old boy when he was first referred to the geneticist, primarily for severe psychomotor retardation and language delay. Physical examination at that time revealed that he had microcephaly with frontal narrowing, a flat philtrum, and bilateral epicanthal folds. His head circumference was 47 cm, which was below three standard deviations from the mean of his age in our population. His height was at the 25th percentile, while his body weight was between the 50th to 75th percentile. No seizure history was detected. He was born spontaneously at full term with an uneventful pregnancy. The details of his birth history were not available beyond that his birth weight was 2800 g. The Chinese-language version of the Denver Developmental Screening Test (DDST) conducted at 6 years old showed that he had severe psychomotor retardation and a mental age of about 2 years. At the age of 9, he was given the Chinese version of the Wechsler Intelligence Scale for Children-Third Edition (WISC-III), which showed that he had moderate MR with a verbal intelligence quotient (IQ) score of 46, performance IQ score of 49, and full IQ score of 47. At the age of 10, a brain magnetic resonance imaging (MRI) analysis of the proband showed no abnormality.

His mother was also found to have severe MR, limited language, and microcephaly. The father was unrelated to the mother, had normal intelligence and had a job. However, he reported that he had a family history of depression. The second boy in this family had normal intelligence and developmental milestones; however, the third boy also suffered from severe psychomotor retardation and microcephaly. At the age of 5 years and 7 months, the third boy had head circumference of 45 cm, which was below the 3rd percentile. The Chinese-language version of the DDST administered at that time also showed that this boy had severe psychomotor retardation and a mental age of about 2 years. He was found to have tics at the age of 7. At the age of 9 years, brain MRI study showed that this boy had hypoplasia of the frontal brain lobes and anterior portion of the corpus callosum. The maternal grandmother of this family was reported to have mental retardation too; and the affected mother had a younger brother who was reported to have normal intelligence. However, neither of them was available for this study.

### Karyotype and FISH analysis

Karyotype analysis of this family showed a reciprocal chromosomal translocation between chromosomes 1p and 12q, t(1;12)(p32.1;q21.3), co-segregating with the affected members in this family (Figure 1B). The father and the second boy, both of whom were normal in intelligence, had normal karyotypes. We conducted FISH analysis to fine map the breakpoint regions of the reciprocal translocation. Two BAC clones that span the respective breakpoints of the translocation on chromosomes 1 and 12 were identified. As shown in Figure 1C, the fluorescent signal of BAC RP11-57F6 (GenBank accession numbers: AQ194822 and AQ115454) was detected in normal chromosome 1, derivative chromosome 1, and derivative chromosome 12, indicating that the chromosome 1 breakpoint was located within the ~ 140 kb genomic sequences of RP11-57F6. Similarly, the fluorescent signal of RP11-259D24 (GenBank access numbers AQ483628 and AZ519051) was present in normal chromosome 12, derivative chromosome 12, and derivative chromosome 1 (Figure 1D), indicating the chromosome 12 breakpoint was located within the ~ 140 kb genomic sequences of RP11-259D24.

### Breakpoint identification

**Figure 2:**
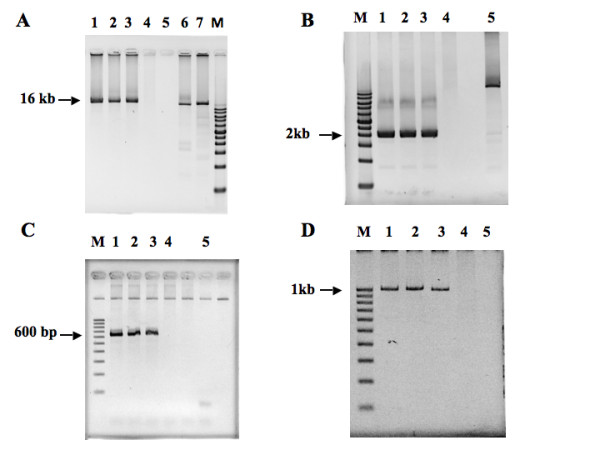
**PCR-based breakpoint identification.** (A) Long-range PCR products of ~ 16 kb covering the breakpoint region of the derivative chromosome 12. M: DNA size marker; 1. II-1, 2. II-3, 3: I-2, 4: I-1, 5: water blank, 6: positive control for long-range PCR using genomic DNA as a template, 7. positive control for long-range PCR using phage lambda as a template. (B) The breakpoint region of derivative chromosome 12 was further narrowed down to a PCR product of ~ 2 kb. M: DNA size marker; 1. II-1, 2. II-3, 3: I-2, 4: I-1, 5: positive control for long-range PCR using genomic DNA as a template (C). To sequence the breakpoint region, a PCR product of 600 bp covering the breakpoint region of derivative chromosome 12 was generated and subjected to autosequencing. M: DNA size marker; 1. II-1, 2. II-3, 3: I-2, 4: I-1, 5: water blank. (D) PCR products of ~ 1 kb covering the breakpoint region of derivative chromosome 1 were obtained and subjected to autosequencing. M: DNA size marker; 1. II-1, 2. II-3, 3: I-2, 4: I-1, 5: water blank.

**Figure 3:**
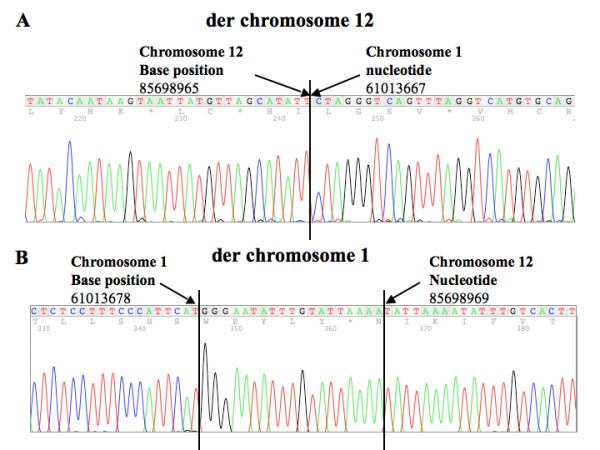
**The sequences of chromosome 1 and chromosome 12 breakpoint.** The sequences flanking the breakpoint junction of derivative chromosome 12 (A) and derivative chromosome 1 (B) were obtained after autosequencing the PCR amplicons covering the breakpoint regions. The nucleotide positions of the breakpoints were based on the human genome sequences Build 37.1.

To identify the breakpoint sequences, we used long-range PCR to obtain amplicons that contained the fusion sequences of derivative chromosome 1 and derivative chromosome 12, respectively. We successfully obtained a PCR amplicon of ~ 16 kb covering the breakpoint of the derivative chromosome 12 from the affected members using the primer Chr 12-5 located on chromosome 12, and the primer Chr 1-6 located on chromosome 1 (Figure 2A). The breakpoint region was further defined using the other internal primer sets. At first, a PCR amplicon of ~ 2 kb was obtained using the internal primers (Chr 12-5-8 and Chr 1-6-6) (Figure 2B); then a PCR amplicon of ~ 600 bp (Figure 2C) was obtained using two other internal primer sets (Chr12-Narrow F and Chr1-Narrow F) (Figure 2C). The ~ 600 bp PCR products were subjected to autosequencing. As shown in Figure 3A, the breakpoint junction was located at nucleotide position 85698965 on derivative chromosome 12 (Human Genome Sequences, Build 37.1) and at nucleotide position 61013667 on chromosome 1 (Human Genome Sequences, Build 37.1); there was a deletion of 3 base pairs (TTG) from derivative chromosome 12 in this fusion sequence.

The determination of the breakpoint sequences on derivative chromosome 12 allowed us to build primer sets (Chr1-Narrow R and Chr12-Narrow R) to conduct PCR to obtain an amplicon of ~ 1 kb that encompassed the breakpoint region on derivative chromosome 1 (Figure 2D). The amplicon was sequenced, and the junction sequences are shown in Figure 3B. The breakpoint junction included nucleotide position 61013678 on derivative chromosome 1 (Build 37.1) and nucleotide position 85698969 on chromosome 12 (Build 37.1); there was an insertion of 19 base pairs in this junction region. All the affected children had the same breakpoint sequences that were transmitted from their mother. The sequences and the locations of the primers used for this molecular analysis of the breakpoints are listed in Table 1.

### Reporter gene assay

**Figure 4:**
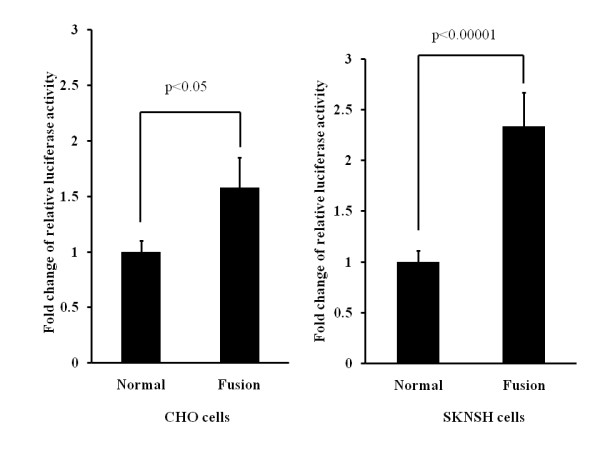
**Reporter gene assays of the fusion sequences from derivative chromosome 12 and corresponding normal chromosome 12 sequences**. (A) In the CHO cells, the construct containing the fusion sequences had luciferase activity about 1.5-fold higher than that of the construct that contained the normal sequences. (B) Similarly, in SKNSH cells, the construct containing the fusion sequences had luciferase activity about two times higher than that of the construct that contained the normal sequences.

In the CHO cells, the fusion construct of chromosomes 12 and 1 had ~ 1.5-fold increased reporter gene activity compared with the corresponding regular chromosome 12 construct, as shown in Figure 4A. In SKNSH cell lines, the same fusion construct had ~ 2.0-fold increased reporter gene activity compared to the corresponding regular chromosome 12 construct, as shown in Figure 4B.

## Discussion

In the present study, we successfully mapped the breakpoint sequences of a reciprocal translocation between chromosome 1 and 12 that co-segregated with mental retardation, language delay, and microcephaly in a two-generation family. The breakpoint on chromosome 1 did not disrupt any known gene directly. The genes closest to this breakpoint included the CYP2J2 gene located ~ 621 Kb centromeric from the breakpoint, and the NFIA gene located ~ 529 kb telomeric from the breakpoint. The chromosome 12 breakpoint did not directly disrupt any known gene, either. The breakpoint was located ~ 3.4 kb downstream of the 3' UTR of the ALX1 gene, which was the gene closest to the breakpoint. The ALX1 gene encodes the aristaless-like homeobox 1 transcription factor that is involved in craniofacial development. Disruption of the ALX1 gene was recently reported to cause an autosomal recessive microphthalmia and a severe facial cleft [14]. In this study, ALX1 was considered as a putative candidate gene associated with the clinical symptoms of this family in view of its close location to the breakpoint and its biological function. We hypothesized that the expression of the ALX1 gene might be changed via the position effect by the translocation [7,15,16]. To test this hypothesis, we first attempted to measure the mRNA level of the ALX1 gene from lymphoblastoid cells in affected family members and control subjects using RT-qPCR; however, the mRNA level of the ALX1 gene was not detectable in the lymphoblastoid cells. To assess the influence of the translocation on ALX1 gene expression, we next conducted a reporter gene assay in Chinese ovary hamster (CHO) cells and a SKNSH neuroblastoma cell line. The results from this assay showed that the fusion construct between chromosome 12 and 1 had significantly higher reporter gene activity than the corresponding normal chromosome 12 sequences, indicating the fusion construct might enhance ALX1 gene expression.

The ALX1 gene, also known as the CART1 gene, encodes a 326-amino acid cartilage paired-class homeoprotein 1 that acts as a transcription factor and binds to palindromic sequences consisting of two TAAT separated by 3 or 4 base pairs [17]. In rodents, the Alx1 gene is expressed in forebrain mesenchymal cells, branchial arches, limb buds, and chondrocytes during embryogenesis [18,19]. Homozygous Alx1 gene knockout mice did not show bone abnormalities, instead, they showed defects in neural tube closures that resulted in acrania, meroanencephaly, and other head abnormalities [19], while the heterozygous Alx1 knockout mice were apparently normal [19]. Recently, Uz and colleagues reported the first human clinical phenotypes associated with ALX1 gene mutation in two families [14]. They found that complete loss of the ALX1 function in these two families seriously disturbed early craniofacial development and resulted in severe frontofacionasal dysplasia, including bilateral extreme microphthalmia, severe facial cleft, complete palate cleft, and low-set posteriorly rotated ears; the frontofacionasal dysplasia in these two families presented in a recessive mode of inheritance. In one family, the affected family members showed a homozygous 3.7 Mb deletion containing the ALX1 gene. In the other family, a homozygous G-to-A mutation at the donor-splicing site of intron 2 was identified in the affected patient. The clinical symptoms caused by the loss-of-function mutations of the ALX1 gene were related to the clinical spectrum of frontofacionasal dysplasia caused by the two other ALX genes, ALX3 and ALX4. Patients with homozygous mutations in the ALX3 gene presented distinctive clinical features of frontofacionasal malformations, including hypertelorism, wide nasal bridge, bifid nasal tip, broad columella, widely separate slit-like nares, long philtrum with prominent bilateral swellings, and midline notch in the upper lip and alveolus, termed frontorhiny [20]. Homozygous mutation in the ALX4 gene was found to be associated with frontofacionasal dystosis syndrome, characterized by total alopecia, a large skull defect, coronal craniosynostosis, hypertelorism, a severely depressed nasal bridge and ridge, a bifid nasal tip, hypogonadism, callosal body agenesis, and mental retardation [21]. Together, these findings suggest all three ALX family genes have an essential, non-redundant role in the craniofacial development in humans, and the loss-of-function mutations of these genes may cause severe cranio-facial-nasal abnormalities.

Based on the reporter gene assay, we speculate that the clinical phenotypes of the family in the present study including severe MR, language delay, and microcephaly are likely to be associated with over-expression of the ALX1 gene, which contrasts with the clinical phenotypes of the loss-of-function mutation of the ALX1 gene as reported recently [14]. The pathogenesis of the over-expression of the ALX1 gene needs further investigation. A previous study reported that the homodimerization of the Alx1 is necessary for its transcription activity [22]. In their study, the authors isolated two additional isoforms of the Alx1 that were generated by alternative splicing from rat chondrosarcoma. These two additional isoforms competed with the wild-type Alx1, exerting a dominant negative effect. Brouwer et al. also reported that expression of mutant Alx1 cDNA with the deletion of the aristaless-domain resulted in severe cranial and vertebral abnormalities, supporting a dominant negative role or gain-of-function of certain mutations in the Alx1 gene [23]. Hence, we speculate that the over-expression of ALX1 might have a dominant negative or gain-of-function effect and result in the clinical phenotypes of this family. The clinical features associated with the over-expression of the ALX1 gene have not yet been reported in the literature; the family reported in the present study might be the first to indicate that increased expression of the ALX1 gene may lead to microcephaly, language delay, and mental retardation. Our findings may expand the clinical spectrum of ALX-related gene mutations in humans.

Notably, the association between the translocation and the clinical presentations in this family should be interpreted with caution. As an alternative, it is likely that the concurrence of the translocation in the affected family members might be due just to chance. In addition, the maternal grandmother of this family was reported to have mental retardation too; and the affected mother had a younger brother who was reported to have normal intelligence. However, neither of them was available for investigation of the origin of the translocation, which is a limitation of this study.

## Conclusion

We identified a reciprocal translocation t(1;12)(p32.1;q21.3) that co-segregates with microcephaly, language delay, and mental retardation in a two-generation family. The mapping and characterization of the breakpoint sequences suggest that the translocation may enhance the gene expression of the ALX1 via the position effect and may thus lead to clinical phenotypes of this family.

## Competing interests

The authors declare that they have no competing interests.

## Authors' contributions

HML conducted the long-range PCR and sequencing; JSF and KFL conducted the karyotype analysis; YJC conducted the FISH analysis; KLW conducted the reporter gene assay; CHC recruited and evaluated the clinical phenotypes of the family and supervised the study; he also wrote the draft of the manuscript that was revised and approved by all the authors.

## Pre-publication history

The pre-publication history for this paper can be accessed here:

http://www.biomedcentral.com/1471-2350/12/70/prepub
